# Development and validation of the internet gaming disorder scale-9 short form Japanese version for children for early screening in elementary school children

**DOI:** 10.3389/frcha.2025.1622000

**Published:** 2025-07-10

**Authors:** Azusa Ogiso, Takeshi Inoue, Tasuku Kitajima, Yuta Ujiie, Yuji Oto, Ryoichi Sakuta

**Affiliations:** ^1^ Child Development and Psychosomatic Medicine Center, Dokkyo Medical University Saitama Medical Center, Saitama, Japan; ^2^ Nagasaki Prefectural Center of Medicine and Welfare for Children, Nagasaki, Japan; ^3^ Department of Psychology, College of Contemporary Psychology, Rikkyo University, Saitama, Japan; ^4^ Department of Pediatrics, Dokkyo Medical University Saitama Medical Center, Saitama, Japan

**Keywords:** internet gaming disorder, child mental health, screening tool, questionnaire validation, elementary school children

## Abstract

**Introduction:**

The increasing use of digital devices has led to growing concern over Internet Gaming Disorder (IGD) among younger children. While several tools for the assessment of IGD have been developed, validated questionnaires have primarily been designed for children aged nine years and older, leaving a gap for early detection. This study developed and validated the Internet Gaming Disorder Scale-9 Short Form Japanese version for Children (IGDS9-SF-JC), a self-reported screening tool tailored for lower elementary school children.

**Methods:**

The IGDS9-SF-JC was developed in collaboration with pediatric neurologists, child and adolescent psychiatrists, clinical psychologists, and elementary school teachers. This study assessed 525 children aged 6–12 years studying at a public elementary school in Chiba Prefecture, Japan.

**Results:**

The IGDS9-SF-JC demonstrated high internal consistency (Cronbach's *α* = 0.849). Confirmatory factor analysis indicated a unidimensional structure with acceptable model fit indices (GFI = 0.942, CFI = 0.931, RMSEA = 0.085). In general, boys had significantly higher total scores than girls, and higher scores were associated with ownership of a gaming device and/or a smartphone, longer times spent gaming and video-watching, later bedtimes, and skipping breakfast. These results are consistent with previous findings of IGD and lifestyle factors in older children and adolescents.

**Discussion:**

The IGDS9-SF-JC expands the applicability of IGD screening to younger children, providing a reliable and valid tool for the early identification and potential intervention of IGD. Further studies are required to refine the instrument and establish clinical cutoff scores using comparison with clinical populations.

## 1 Introduction

With the widespread use of digital devices, gaming has become one of the most easily accessible and popular forms of entertainment. Although gaming can enhance cognitive functions and promote positive emotions (1), excessive gaming reportedly has a potential negative impact on mental health.

Problematic gaming behavior has been considered as Internet Gaming Disorder (IGD) under “Conditions for Further Study” in the Diagnostic and Statistical Manual of Mental Disorders, 5th Edition (DSM-5) (2). According to DSM-5, IGD has nine diagnostic criteria: (1) Preoccupation, (2) withdrawal, (3) tolerance, (4) unsuccessful attempts to control, (5) loss of interest in other activities, (6) continued use despite negative consequences, (7) deceptiveness, (8) escape from problems, and (9) jeopardizing relationships or opportunities. An individual is diagnosed with IGD when five or more of these symptoms persist for at least 12 months. In 2019, IGD was officially included in the International Classification of Diseases, 11th Revision (ICD-11) (3), under the name Gaming Disorder (GD) in the category of “Disorders due to substance use or addictive behaviors.” The diagnostic criteria for GD in ICD-11 include (1) impaired control over gaming, (2) prioritization gaming over other activities, and (3) continuation or escalation of gaming despite negative consequences.

Additionally, these behavioral patterns should result in significant impairment in functioning for at least 12 months before GD diagnosis is made. While the conceptual differences between IGD and GD remain debatable, both share the following core characteristics: difficulty in controlling gaming behavior and prolonged gaming despite associated problems. These features are clinically significant because they lead to severe functional impairment.

Recently, concerns regarding problematic gaming and internet use among younger children have been raised (4). A meta-analysis of 36 studies conducted in Southeast Asia (5) reported an IGD prevalence rate of 9.3% (confidence interval: 8.2%–10.5%) among children and adolescents. Although data on IGD prevalence in Japan remain limited, we have noticed an increasing number of reports from medical institutions involving elementary and junior high school students experiencing difficulty waking up caused by prolonged use of the internet, gaming consoles, and smartphones, leading to repeated tardiness and absenteeism (6). In the same report, a survey involving 11,086 elementary school students and their parents found that 19.4% of lower-grade boys and 10.6% of lower-grade girls met the DSM-5 diagnostic criteria for IGD.

Although research on the negative impact of IGD in children is still in progress, IGD has been associated to several factors, including depressive symptoms (7), anxiety and social phobia (8), academic underachievement (9), sleep disorders (10), and lifestyle disruptions, e.g., skipping breakfast (11). Thus, further research in this field is urgently needed.

Questionnaires are commonly used for assessing and screening IGD, and various psychometric tools have been developed to establish a consensus on scientific findings and enable a standardized approach. A systematic review by (12) examining 32 IGD assessment tools identified several questionnaires with relatively high levels of empirical evidence. These questionnaires include the Game Addiction Scale (GAS-7) (13), Internet Gaming Disorder Scale-9-item Short Form (IGDS9-SF) (14), Internet Gaming Disorder Test-10-item (IGDT-10) (15), Young's Diagnostic Questionnaire (16), and Internet Gaming Disorder Scale-9-item (Lemmens IGD-9) (17). Among them, IGDS9-SF has been translated into 17 languages, making it the most widely adopted tool. It allows for a simple screening of IGD risk according to the nine DSM-5 criteria (18). Its measurement invariance across gender, structural validity, and internal consistency have been extensively validated (18, 19), and its Japanese version was developed and standardized by (20).

However, existing standardized IGD questionnaires are designed for children aged nine years and older only (20, 21), with no validated tools currently available for younger elementary school children. Considering that younger children generally have underdeveloped ability to understand abstract concepts, questionnaire-based assessments are commonly administered to children in the upper elementary grades and beyond. Despite the challenges of developing assessment tools for younger children, research has revealed that early exposure to online gaming before school age may increase the risk of IGD (21). Therefore, a lower-age version (child version) of an IGD assessment tool must be developed to expand the target population, facilitate further research on GDs, and support earlier screening for preventive interventions.

Unlike other studies, this study developed a questionnaire that can be applied to lower elementary school children for assessing GD, a condition that has been globally recognized to increasingly affect younger populations. The specific objectives of this study were as follows: (1) development of a questionnaire applicable to younger children (child version) on the basis of the Internet Gaming Disorder Scale-9 Short Form Japanese version (IGDS9-SF-J), which has established empirical evidence internationally and has been standardized in Japan, and (2) examination of the validity and reliability of the newly developed tool that is, the IGDS9-SF-J child version (IGDS9-SF-JC), and evaluation of its utility.

## 2 Materials and methods

### 2.1 Development of the IGDS9-SF-JC

Three experts—a board-certified pediatric neurologist, a board-certified child-and-adolescent psychiatrist, and a clinical psychologist—independently developed the IGDS9-SF-JC by referring to both the original IGDS9-SF (14) and the Japanese version IGDS9-SF-J (20) a 9-item self-reported questionnaire with each item scored from 1 to 5 (total score: 9–45). The questionnaires created by each expert were reviewed and revised from semantic, idiomatic, experiential, and conceptual perspectives. Subsequently, two elementary school teachers and a pediatric neurologist evaluated the questionnaire's appropriateness, leading to further modifications and the final development of the IGDS9-SF-JC.

### 2.2 Participants and the procedure

The study was conducted in Nagareyama City located in Chiba Prefecture in Japan. This city met the following criteria: (a) a medium-sized city with a population of approximately 150,000–250,000, (b) a suburban area with a mix of urban and rural characteristics, and (c) situated approximately 0.5 h from the Tokyo metropolitan area. All 590 students aged 6–12 years who attended a centrally located public elementary school A within Nagareyama City were selected as participants.

The instrument validation study was conducted between November 15 and December 12 of 2023. The survey included the following components: IGDS9-SF-JC, demographic information (e.g., age and sex), electronic device ownership, electronic device usage patterns, gaming behavior, lifestyle habits (e.g., wake-up and bedtime routines), and school-related factors (e.g., absences and study time). The battery of questionnaires was administered online using school-issued tablets during the homeroom periods, under the supervision of the classroom teachers. Initially, the participants were instructed to answer all questions honestly and to avoid spending excessive time on any single question.

### 2.3 Statistics

Of the 554 respondents (93.9%), 29 (4.9%) provided inappropriate responses. Consequently, data from 525 respondents (89.0%) were analyzed.

Findings with a *p*-value less than 0.05 were considered statistically significant. The questionnaire's internal reliability was assessed by calculating the Cronbach's alpha (α). Confirmatory factor analysis (CFA) and model fit evaluation were conducted using the lavaan package (22) in R (version 4.4.1) with RStudio (version 2024.04.2). For CFA, multiple goodness-of-fit indexes were used: goodness-of-fit index (GFI), comparative-of-fit index (CFI), normed fit index (NFI), adjusted goodness-of-fit index (AGFI), root mean square error of approximation (RMSEA), and standardized root mean square residuals (SRMR). GFI, CFI, NFI, and AGFI values of 0.90 or higher are considered indicative of good model fit, SRMR value of 0.08 or lower also indicates a good fit (23). For RMSEA, a value of 0.05 or lower suggests a good fit, values between 0.05 and 0.08 indicate a reasonable fit, values between 0.08 and 0.10 suggest a mediocre fit, and values above 0.10 indicate a poor fit (24). Furthermore, the Mann–Whitney *U*-test, Kruskal–Wallis test, and Spearman's correlation analysis were conducted using GraphPad Prism (version 9.5.0).

### 2.4 Ethical considerations

The study protocol obtained approval from the Ethics Committee of the Dokkyo Medical University Saitama Medical Center (No. 22042). Permission to conduct the survey at Public School A was obtained from the school's principal and the superintendent of the Board of Education of Nagareyama City. Additionally, an informational document detailing the study's purpose and measures for protecting personal information was distributed to all households. Only students who provided assent and whose parents gave informed consent were included in this study.

## 3 Results

### 3.1 Developed questionnaire

Table 1 presents the IGDS9-SF-JC, along with the mean and standard deviation (SD) for each item. Compared with the IGDS9-SF-J, the IGDS9-SF-JC uses a simpler vocabulary to accommodate younger children (e.g., obsession → really like, failure as a plan → not doing well, betray → disappoint). Additionally, examples were added to items 5, 6, 7, and 8 to enable lower elementary school students to fully understand the questions. The mean total score across all items was 16.61 (SD, 6.34), with a median of 15.5 (IQR, 11.0–20.0) (Table 1).

**Table 1:** Developed questionnaire items and their mean and standard deviation.

Questionnaire Item	Mean	SD^a^
1. Do you really like playing games and think about games a lot? (Example: You often think about games./You feel that playing games is very important.）	2.60	1.20
2. Do you feel frustrated or sad when you try to reduce your game time or stop playing games?	1.89	1.11
3. Do you wish you had more time to play games?	2.43	1.33
4. Even if you make rules about games or try to stop, is it hard to follow the rules?	1.99	1.13
5. Did you stop doing fun things you used to like because playing games is so fun? (Example: You don't play at the park or make crafts anymore.)	1.74	1.07
6. Even when playing games causes problems, do you keep playing? (Example: Even if you can't wake up in the morning because of games, you still keep playing.)	1.55	0.96
7. Has playing too many games made your family, friends, or teachers feel disappointed? (Example: You forgot to meet a friend, or you kept playing even after the time you promised to stop.)	1.43	0.72
8. When you feel bad, do you sometimes play games to forget those feelings? (Example: You feel frustrated because you don't want to do homework, or you feel upset after a fight with a friend, so you play games.)	1.74	1.08
9. Because of games, have you ever fought with your family or friends, or skipped school or lessons?	1.22	0.60
all item	16.61	6.34
all item median (IQR)^b^	15.5 (11.0-20.0)	

^a^ SD, standard deviation.

^b^ IQR, interquartile rangE.

### 3.2 Descriptive statistics

Table 2 summarizes the sociodemographic characteristics of the participants. These participants had a mean age of 9.87 ± 1.85 years (range: 6–12 years), with a sex ratio of approximately 1:1. Among the 525 participants, 452 (86.1%), 335 (63.8%), and 241 (45.9%) reported owning a gaming device, a tablet, and a personal smartphone, respectively. Moreover, 16.8% played games for more than 3 h per day on weekdays and 31.1% on weekends. On the other hand, 88 participants (16.8%) reported not playing games at all on either weekdays or weekends.

**Table 2:** Socio-demographic characteristics of the participants.

Variable	Response option(s)	*n*	%
N		525	
Average age (SD)	9.87 (1.85)		
Sex	Male Female Prefer not to answer	258 254 13	49.1 48.4 2.5
Ownership of game device	Yes No	452 73	86.1 13.9
Ownership of tablet	Yes no	335 190	63.8 36.2
Ownership of smartphone	Yes no	241 284	45.9 54.1
Wake-up time	–7:00 7:00–8:00 8:00–9:00 9:00–10:00 10:00– Don't know	412 87 2 3 0 21	78.5 16.6 0.4 0.6 0.0 4.0
Bed time	–21:00 21:00–22:00 22:00–23:00 23:00–24:00 24:00– Don't know	102 266 102 25 11 19	19.4 50.7 19.4 4.8 2.1 3.6
Breakfast habit	Eat Sometimes eat Don't eat	493 26 6	93.9 5.0 1.1
Study time on weekdays	0 in 30 min 1 h 2 h 3 h 4 + hr	28 281 143 39 19 15	5.3 53.5 27.2 7.4 3.6 2.9
school absence (past month)	0day 1–3day 4–6day 7 + day Don't know	332 107 17 12 57	63.2 20.4 3.2 2.3 10.9
Game playing time (weekdays)	0 min 30 min 1 h 2 h 3 h 4 h 5 h 6 + hr	135 111 124 67 46 16 11 15	25.7 21.1 23.6 12.8 8.8 3.0 2.1 2.9
Game playing time (weekend)	0 min 30 min 1 h 2 h 3 h 4 h 5 h 6 + hr	88 84 112 78 54 33 22 54	16.8 16.0 21.3 14.9 10.3 6.3 4.2 10.3
Video watching time (weekdays)	0 min 30 min 1 h 2 h 3 h 4 h 5 h 6 + hr	123 144 117 60 40 16 9 16	23.4 27.4 22.3 11.4 7.6 3.0 1.7 3.0
Video watching time (weekend)	0 min 30 min 1 h 2 h 3 h 4 h 5 h 6 + hr	94 110 115 63 49 32 14 48	17.9 21.0 21.9 12.0 9.3 6.1 2.7 9.1

### 3.3 Reliability analysis and CFA

Regarding the internal consistency of the questionnaire, the Cronbach's alpha was 0.849 across all the nine items of the IGDS9-SF-JC, indicating high reliability. In terms of the construct validity of the questionnaire, CFA was conducted with maximum likelihood (ML) method, under a one-factor model assumption for the nine items of the IGDS9-SF-JC. The model fit indices were as follows: *χ*^2^(27, *N* = 525) = 134.48, *p* < .001; goodness-of-fit index (GFI) = 0.942; comparative fit index (CFI) = 0.931; normed fit index (NFI) = 0.916; adjusted goodness-of-fit index (AGFI) = 0.903; root mean square error of approximation (RMSEA) = 0.085; and standardized root mean square residual (SRMR) = 0.049. GFI, CFI, NFI, and AGFI were all above 0.90, while SRMR was below 0.08, indicating a good model fit. Although the RMSEA value of 0.085 was not ideal, it remained within the acceptable range. Factor loadings for the nine items were 0.48–0.72, demonstrating moderate-to-strong factor loadings (Table 3). Additionally, all factor loadings were statistically significant. These results suggest that the one-factor structure for the IGDS9-SF-JC is valid.

**Table 3:** Standardized factor loadings for the One-factor model.

Item	Standardized factor loading
Item 1	0.69*
Item 2	0.72*
Item 3	0.68*
Item 4	0.60*
Item 5	0.67*
Item 6	0.67*
Item 7	0.52*
Item 8	0.61*
Item 9	0.48*

* *p* < .001 for all loadings.

### 3.4 Group comparisons and correlations with external criteria

The IGDS9-SF-JC total scores were compared between groups based on sex, electronic device ownership, and breakfast consumption. Table 4 summarize the results. The Mann–Whitney *U*-test showed that boys had significantly higher total scores than girls (*p* = .0001). Furthermore, children who owned a gaming device had higher total scores than those who did not (*p* < .0001). Similarly, children who owned a smartphone had higher total scores than those who did not (*p* = .0272). However, no significant difference was observed between the total scores of tablet owners and nonowners (*p* = .382).

**Table 4:** Comparison of IGDS9-SF-JC total score.

Variable	Group	*n*	median (IQR)	*p*-value		
				1 vs. 2	1 vs. 3	2 vs. 3
Sex				0.0001***		
	1. Male	258	17.0 (13.0–21.0)			
	2. Female	254	15.0 (11.0–19.0)			
Ownership of game device				<.0001***		
	1. Yes	452	16.0 (12.0–21.0)			
	2. No	73	11.0 (9.0–16.0)			
Ownership of tablet				0.382		
	1. Yes	335	15.0 (11.0–20.0)			
	2. No	190	16.0 (11.0–21.0)			
Ownership of smartphone				0.0272*		
	1. Yes	241	16.0 (12.0–21.0)			
	2. No	284	15.0 (11.0–19.3)			
Breakfast habit				0.488	0.001**	0.005**
	1. Eat	493	15.5 (11.0–20.0)			
	2. Sometimes eat	26	15.0 (12.0–22.0)			
	3. Don't eat	6	30.0 (24.0–35.3)			

*** *p* < .001.

** *p* < .01.

* *p* < .05.

Regarding breakfast consumption, a Kruskal–Wallis test revealed significant differences among children who did not eat breakfast, those who ate regularly, and those who ate occasionally (*p* = .002). In *post hoc* multiple comparisons using the Bonferroni method, children who did not eat breakfast had higher total scores than those who ate regularly (*p* = .001) and those who ate occasionally (*p* = .005).

Next, a correlation analysis between the IGDS9-SF-JC total scores and other variables, including age, wake-up time, bedtime, study time, number of absences, gaming time, and video-watching time, was conducted. The total scores showed a moderate positive correlation with weekday gaming time (*r* = .476, *p* < .0001) and weekend gaming time (*r* = .491, *p* < .0001). Conversely, the total scores exhibited a weak positive correlation with weekday video-watching time (*r* = .266, *p* < .0001), weekend video-watching time (*r* = .298, *p* < .0001), and bedtime (*r* = .275, *p* < .0001). Age (*r* = .186), wake-up time (*r* = .146), study time (*r* = .132), or the number of absences (*r* = .056) showed no significant correlations with the total scores.

## 4 Discussion

The study developed the IGDS9-SF-JC and evaluated its reliability and validity. Self-administered psychological questionnaires for lower elementary school children are scarce, primarily because younger children have underdeveloped ability to understand abstract concepts. However, some studies have attempted to develop questionnaires applicable for younger children by incorporating specific modifications (23, 24). While constructing questionnaires for younger children, the following points must be considered: designing items based on real-life experiences, using simple and concrete expressions that do not require abstract thinking, limiting the number of items, providing relatable examples, and incorporating oral explanations during administration. The IGDS9-SF-JC was initially developed by physicians and psychologists with expertise in pediatric clinical practice and child development and then revised in collaboration with elementary school teachers. Similar to previous studies, we employed colloquial and simplified language, as well as concrete examples, so that the younger children can understand the questions easily. Additionally, the questionnaire has only nine items, and the responses were collected under the supervision of school teachers, which likely contributed to obtaining valid responses. However, the presence of teacher supervision may have introduced potential bias. For instance, children may have been influenced by social desirability and attempt to provide responses they believed to appropriate or expected in the presence of an authority figure. Therefore, greater caution is needed in interpreting the data, taking into account the context in which the questionnaire was administered.

Regarding reliability, the nine-item questionnaire demonstrated strong internal consistency, with Cronbach's alpha exceeding 0.8. A systematic review reported that all 21 included studies supported the unidimensional structure of IGDS9-SF (18). We conducted CFA assuming a one-factor model, consistent with previous research, and obtained an overall acceptable model fit. Factor loadings ranged from 0.48 to 0.72, indicating moderate-to-high loadings, consistent with the findings of (14), who originally developed IGDS9-SF. These results indicate that the one-factor structure is valid for the lower-age version.

For criterion-related validity, we conducted group comparisons according to demographic factors and device ownership, and correlation analyses between total scores and lifestyle variables. Regarding sex differences, boys had significantly higher total scores than girls, consistent with prior research (25–27). Therefore, even at a young age, males may be more strongly associated with IGD risk. In contrast, age showed no correlation with total scores, aligning with the results of (25). IGD development may result from an interaction of internal factors, such as deficits in self-regulation, mood regulation, reward processing, and decision-making, and external factors, including family background and social skill deficits (4). Therefore, a direct association between older age and higher IGD scores is not necessarily expected in childhood.

Moreover, the total scores positively correlated with gaming time, consistent with the findings of (14). A novel finding of this study was the correlation between total scores and video-watching time. To our knowledge, this association has not been reported elsewhere, making this finding a new contribution to the literature. One possible explanation for this finding is that children with IGD tendencies may also watch gaming-related videos, suggesting a direct link. Other reasons might be the idea that both gaming and video-watching are forms of screen time and that confounding factors such as escapism from real-life stressors and negative emotions may underlie this correlation.

Our results also agree with previous studies on the relationship between gaming behavior and lifestyle habits (26). Reported that students with IGD tend to have more sleep disturbances than those without. Our study found that higher total scores were associated with later bedtimes, suggesting a similar pattern in younger children. Additionally, the total scores were higher in children who skipped breakfast than in those who ate breakfast regularly, consistent with previous research (11). Therefore, children with more problematic gaming behaviors may tend to have more lifestyle-related difficulties.

Furthermore, the total scores were not significantly associated with study time. While previous studies reported that IGD negatively correlates with academic performance (27), our study only assessed impact on study time and did not evaluate effect on academic achievement. Future research should investigate the direct relationship between IGD and academic performance in lower elementary school children. Additionally, the IGD scores were not associated with absenteeism. However, as this study was conducted in a school setting, selection bias may have occurred, given that children who had already refused to attend school may not have been included in the survey. Taken together, the comparisons and correlations with external criteria generally align with previous studies, supporting the questionnaire's reliability and validity.

Considering that the study was conducted in a single elementary school, future research should expand the survey region and involve larger questionnaire investigations. Although the newly developed questionnaire demonstrated acceptable reliability and validity, further refinement is needed to make it more useful as an IGD screening tool. Specifically, future studies should determine the cutoff scores by examining the clinical and nonclinical groups, and investigate the characteristics of gender-specific response patterns. Furthermore, it is essential to investigate whether the diagnostic criteria for gaming disorder, originally developed for adults, are appropriate for use with younger children in lower grades, considering developmental differences.

In conclusion, the IGDS9-SF-JC possesses adequate validity and reliability, making it a useful tool for assessing IGD in lower elementary school children.

## Data Availability

The data analyzed in this study is subject to the following licenses/restrictions: the datasets generated and analyzed during the current study are not publicly available due to the lack of participant consent for secondary use of the data. Requests to access these datasets should be directed to Takeshi Inoue, tinoue@dokkyomed.ac.jp.
